# Risk factors for methicillin-resistant *Staphylococcus aureus* and *Pseudomonas aeruginosa* community-acquired pneumonia at a high-volume transplant center

**DOI:** 10.1017/ash.2026.10849

**Published:** 2026-09-09

**Authors:** Evan K. Mallory, Drew T. Dickinson, Dylan Kosaski, Jacob Schwarz, Emily Thompson, Sandhya R. Nagarakanti, M. Teresa Seville, Dan Ilges

**Affiliations:** 1 Department of Pharmacy, Mayo Clinic Arizona, Phoenix, USA; 2 Division of Infectious Diseases, Mayo Clinic Arizona, Phoenix, USA; 3 Division of Clinical Trials and Biostatistics, Mayo Clinic Arizona, Phoenix, USA; 4 Infectious Diseases, Mayo Clinic Arizona, Phoenix, USA

## Abstract

**Objective:**

To validate risk factors for methicillin-resistant *Staphylococcus aureus* (MRSA) and *Pseudomonas aeruginosa* in patients hospitalized with community-acquired pneumonia (CAP).

**Design:**

Retrospective, single-center, cohort study from October 1, 2018, to March 31, 2023.

**Setting:**

Mayo Clinic Hospital in Phoenix, AZ.

**Participants:**

Adult patients with CAP, identified using ICD-10 diagnosis codes, receipt of systemic antibiotics for a “respiratory tract infection” within 48 hours of hospitalization, and availability of respiratory cultures.

**Methods:**

Multivariate logistic regression was used to identify factors independently associated with MRSA or *P. aeruginosa* isolation in respiratory cultures.

**Results:**

A total of 1,485 patients met inclusion criteria; 32 (2.2%) had MRSA and 81 (5.5%) had *P. aeruginosa* isolated from respiratory culture. Multivariate logistic regression analyses identified substance use disorder [aOR 8.29, *P* < .001], influenza [aOR 7.64, *P* = .003], lung abscess/empyema [aOR 4.58, *P* = .031], and peptic ulcer disease (PUD) [aOR 4.09, *P* = .017] as MRSA risk factors. Bronchiectasis [aOR 3.67, *P* < .001], PUD [aOR 3.48, *P* = .006], and COPD [aOR 2.60, *P* = .005] were independently associated with *P. aeruginosa*. Immunocompromised conditions, including transplantation (solid organ and hematopoietic stem cell) and malignant tumor were not independently associated with either organism.

**Conclusions:**

Our results substantiate previously demonstrated risk factors for MRSA and *P. aeruginosa* in CAP. We did not observe an independent association between immunosuppressed conditions evaluated and isolation of MRSA or *P. aeruginosa*.

## Introduction

Antimicrobial stewardship program (ASP) efforts remain challenged by the empiric use of broad-spectrum antimicrobials (BSAs) in patients hospitalized with community-acquired pneumonia (CAP). Despite the low prevalence of methicillin-resistant *Staphylococcus aureus* (MRSA) and *P. aeruginosa*, antimicrobial use targeting these organisms remains disproportionately high relative to their prevalence in CAP.^1–3^ The Centers for Disease Control and Prevention has identified unnecessary MRSA and *P. aeruginosa* coverage in CAP as a top priority for hospital ASPs.^4^ In 2019, the Infectious Diseases Society of America (IDSA) and American Thoracic Society (ATS) retired the healthcare-associated pneumonia (HCAP) framework introduced in 2005.^5,6^

Guideline recommendations limit empiric anti-MRSA and antipseudomonal therapies to inpatients with established risk factors. The two risk factors provided by IDSA/ATS are (1) prior respiratory isolation of either organism and (2) hospitalization with receipt of intravenous antibiotics within the previous 90 days for those with severe pneumonia.^5^ Beyond these risk factors, IDSA/ATS recommend that hospitals evaluate local epidemiology and validate institution-specific risk factors.

In the absence of institution-specific validation, prescribers may favor BSAs, particularly for immunocompromised patients who were not represented in the IDSA/ATS guideline.^5^

In this study, we aimed to validate risk factors associated with MRSA and *P. aeruginosa* growth in respiratory cultures among patients presenting with CAP at an academic medical center with a high proportion of immunocompromised patients.

## Methods

### Study design

We performed a single-center, retrospective cohort study of patients hospitalized with CAP at Mayo Clinic Hospital in Arizona between October 1, 2018, and March 31, 2023. Mayo Clinic Hospital is a 368-bed academic medical center that performed 4,348 solid organ (SOT) and 1,181 hematologic stem cell transplants (HSCTs) between 2018 and 2023. SOT included heart, kidney, pancreas and liver; lung and laryngeal transplants were not performed during the study period.

### Participants

We screened adult patients with an *International Classification of Diseases—10^th^ revision* (ICD-10) discharge diagnosis code for pneumonia. We included those who had respiratory cultures and received systemic antibiotics for a “respiratory tract infection” within 48 hours of admission. Our institution requires all inpatient antimicrobial orders to include an order-specified indication at the time of entry. Prior studies demonstrated this approach to have a high degree of accuracy for antibiotic indication.^7,8^

We excluded those who did not have respiratory culture data. We excluded patients who transferred from outside facilities and those with respiratory cultures positive for MRSA or *P. aeruginosa* within the 12 months prior to the CAP admission.

### Data collection

All data were extracted from the electronic health record and an institutional data warehouse. Baseline demographics, including age, sex, race, ethnicity, and transplant history, were collected. Most risk factors were identified using the Charlson Comorbidity Index (CCI), as previously described.^9,10^ Additional risk factors were collected using the following ICD-10 codes: primary immunodeficiency disorders (D71, D80, D81, D82, D83, D84, D89), bronchiectasis (J47), HIV (Z21), influenza (J09, J10, J11), substance use disorder (F19, F11, F14, F55, F15), alcohol use disorder (F10), tracheostomy (Z93.0), wound care (Z48.00).

### Outcomes

The primary objective was to identify factors associated with MRSA or *P. aeruginosa* isolation in respiratory cultures among hospitalized patients with CAP.

### Data analysis

For the primary analysis, multivariate logistic regression was used to calculate an adjusted odds ratio (aOR) for the association between each risk factor and respiratory isolation of MRSA or *P. aeruginosa*. Separate models were constructed for each organism. Three sequential models were run for each risk factor as follows:

1. Unadjusted/univariate model (infection as the outcome and risk factor as the predictor)
2. Model for each risk factor adjusted for age, sex, and BMI (confounder-adjusted)
3. Multivariate model adjusted for age, sex, BMI, and any risk factors found in step 2 to have a *P*-value ≤ .1.

Variables with zero event counts in at least one group were considered not estimable and are reported as such.

In a secondary analysis, risk factors for ICU admission, length of stay, 30-day readmission, and 30-day mortality were assessed using the same covariates as the primary analysis, with additional adjustment for MRSA or *P. aeruginosa* isolation. Logistic regression was used for ICU admission, 30-day readmission, and 30-day mortality, while length of stay (hours) was modeled using Gamma regression with a log link. Statistical significance was defined as a two-sided *P*-value < .05.

## Results

### Population

**Figure 1.**
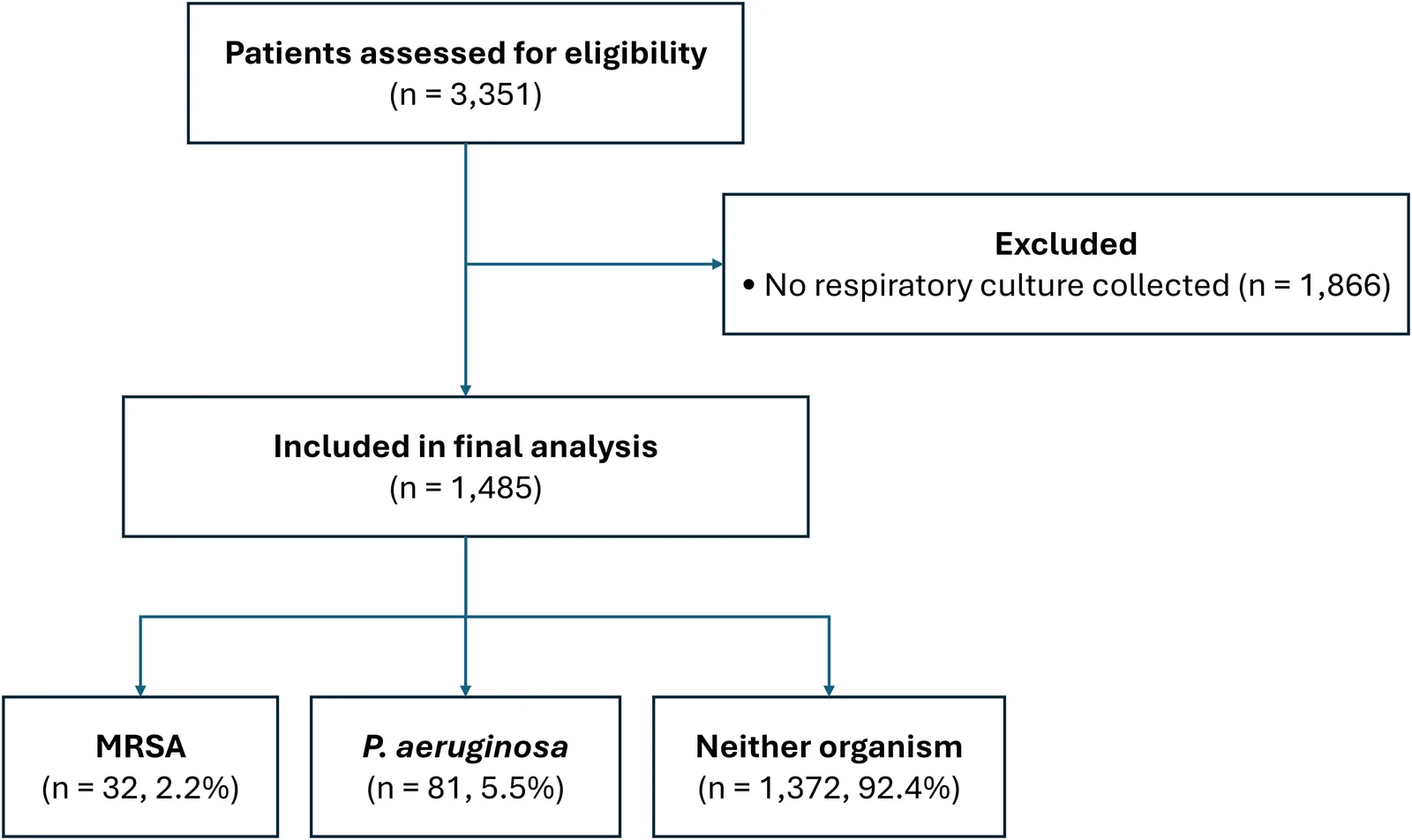
Flowchart for patients hospitalized with CAP at Mayo Clinic Arizona between october 1, 2018, and march 31, 2023.

A total of 3,351 patients were screened for inclusion (admission for CAP diagnosis with receipt of antibiotics with respiratory tract infection indication within 48 h of admission). Of these, 1,866 were excluded due to no respiratory culture collected (Figure 1). This resulted in a final cohort of 1,485 patients, reflecting a severely ill CAP population, with 618 (41.6%) requiring admission to the intensive care unit (ICU), 287 (19.3%) requiring mechanical ventilation, and 215 (14.5%) dying within 30 days of admission (see Table 1 for baseline characteristics).

**Table 1.** Characteristics of patient population Table 1 long description.

	Total (*n* = 1,485)
Age at admission median (IQR)	68 (57, 77)
Male sex, *n* (%)	916 (61.7%)
BMI median (IQR)	25.7 (22.3, 30)
Race, *n* (%)	
American Indian/Alaskan Native	18 (1.2%)
Asian	49 (3.3%)
Black or African American	50 (3.4%)
Hispanic or Latino	128 (8.6%)
Other	11 (0.7%)
White	1,229 (82.8%)
Congestive heart failure	475 (32%)
Peripheral vascular disease	653 (44%)
Cardiovascular disease	265 (17.8%)
Diabetes mellitus	358 (24.1%)
Renal disease	462 (31.1%)
Liver disease	328 (22.1%)
COPD	554 (37.3%)
Bronchiectasis	108 (7.3%)
Lung abscess / empyema	32 (2.2%)
PUD	77 (5.2%)
Rheumatologic disease	109 (7.3%)
Hemiplegia	49 (3.3%)
Dementia	51 (3.4%)
Cancer	568 (38.2%)
Metastatic solid tumor	244 (16.4%)
AIDS	11 (0.7%)
Primary immune deficiency diseases	425 (28.6%)
Hypogammaglobulinemia	57 (3.8%)
Alcohol use disorder	82 (5.5%)
Substance use disorder	61 (4.1%)
Tobacco use	32 (2.2%)
COVID-19	129 (8.7%)
Influenza	35 (2.4%)
Any transplant	240* (16.2%)
HSCT	70 (4.7%)
Any SOT	173 (11.6%)
Heart transplant	47 (3.2%)
Liver transplant	41 (2.8%)
Kidney transplant	107 (7.2%)
Pancreas transplant	5 (0.3%)
Multiple SOT	27 (1.8%)
SOFA score, median (IQR)	9 (6, 12)
APACHE score, median (IQR)	84 (64, 108.8)
ICU admission	618 (41.6%)
Mechanical ventilation	287 (19.3%)
Tracheostomy	174 (11.7%)
Neutropenia (ANC < 500)	14 (0.9%)
30-day readmission	326 (22%)
*3 patients had a history of both a BMT and SOT.	

Regarding immunosuppression, 173 (11.6%) had undergone SOT (107 kidney, 47 heart, 41 liver, and 5 pancreas), with 27 (1.8%) having undergone >1 SOT. Seventy patients (4.7%) had undergone HSCT. 568 (38.2%) had a cancer diagnosis and 244 (16.4%) had metastatic solid tumors. 425 (28.6%) had primary immunodeficiency condition, including 57 (3.8%) with hypogammaglobulinemia, and 14 (.9%) had neutropenia.

Thirty-two (2.2%) patients had MRSA and 81 patients (5.5%) had *P. aeruginosa* isolated from respiratory culture.

Variables evaluated in the multivariate logistic regression models are presented in Tables 2 and 3; complete univariate analyses are provided in Supplementary Appendix. Multivariate logistic regression analysis for MRSA CAP is shown in Table 2. MRSA CAP was associated with substance use disorder [aOR 8.29, 95% CI (2.55–27.0)] (*P* < .001), influenza [aOR 7.64, 95% CI (2.01–29.1)] (*P* = .003), lung abscess/empyema [aOR 4.58, 95% CI (1.15–18.2)] (*P* = .031), and peptic ulcer disease (PUD) [aOR 4.09, 95% CI (1.29–12.9)] (*P* = .017). Alcohol use disorder was associated with MRSA in unadjusted analysis but did not remain significant in the multivariate logistic regression analysis [aOR 2.85, 95% CI (.87–9.27)] (*P* = .082).

**Table 2.** Risk factors for patients with MRSA pneumonia Table 2 long description.

Characteristic	Unadjusted		Adjusted for age, sex, BMI		Adjusted for age, sex, BMI and significant risk factors	
	OR (95% CI)	*P*-value	OR (95% CI)	*P*-value	OR (95% CI)	*P*-value
Lung abscess / empyema	5.05 (1.56–16.32)	.007	4.22 (1.20–14.86)	.025	**4.58 (1.15–18.19)**	**.031**
PUD	3.08 (1.10–8.66)	.033	2.84 (0.96–8.44)	.060	**4.09 (1.29–12.94)**	**.017**
Alcohol use disorder	3.13 (1.21–8.11)	.019	2.85 (1.02–7.95)	.046	2.85 (0.87–9.27)	.082
Substance use disorder	5.49 (2.21–13.63)	<.001	5.45 (1.91–15.60)	.002	**8.29 (2.55–26.97)**	**<.001**
Influenza	6.31 (2.18–18.25)	<.001	5.58 (1.82–17.08)	.003	**7.64 (2.01–29.09)**	**.003**

Abbreviations: CI = confidence interval, OR = odds ratio.

**Table 3.** Risk factors for patients with *P. aeruginosa* pneumonia Table 3 long description.

Characteristic	Unadjusted		Adjusted for age, sex, BMI		Adjusted for age, sex, BMI and significant risk factors	
	OR (95% CI)	*P*-value	OR (95% CI)	*P*-value	OR (95% CI)	*P*-value
Renal disease	0.55 (0.32–0.97)	.040	0.53 (0.30–0.95)	.033	0.63 (0.33–1.19)	.153
Liver disease	0.40 (0.19–0.83)	.014	0.41 (0.19–0.88)	.022	**0.39 (0.17–.92)**	**.030**
COPD	3.02 (1.82–5.01)	<.001	2.84 (1.69–4.77)	<.001	**2.60 (1.34–5.05)**	**.005**
Bronchiectasis	6.43 (3.76–11.01)	<.001	5.11 (2.86–9.13)	<.001	**3.67 (1.85–7.28)**	**<.001**
Lung abscess / empyema	2.58 (0.93–7.19)	.070	2.55 (0.85–7.62)	.094	1.72 (0.47–6.36)	.415
PUD	2.70 (1.31–5.57)	.007	2.50 (1.18–5.30)	.017	**3.48 (1.44–8.44)**	**.006**

Abbreviations: CI = confidence interval, OR = odds ratio.

Multivariate logistic regression analysis for *P. aeruginosa* CAP is shown in Table 3. *P. aeruginosa* CAP was independently associated with bronchiectasis [aOR, 3.67; 95% CI (1.85–7.28)] (*P* < .001), PUD [aOR 3.48, 95% CI (1.44–8.44)] (*P* = .006), and COPD [aOR 2.60, 95% CI (1.34–5.05)] (*P* = .005). Liver disease was found to have a negative association with *P. aeruginosa* [aOR .39, 95% CI (.17–.92)] (*P* = .030).

In our secondary analyses, MRSA isolation was independently associated with longer hospital length of stay. Patients with MRSA had hospital stays that were 1.58 times longer (95% CI, 1.13–2.29, *P* = .010) than those without MRSA after multivariate adjustment. We did not find MRSA or *P. aeruginosa* to be significant risk factors for ICU admission, 30-day readmission, or 30-day mortality.

## Discussion

We did not find transplantation (SOT and HSCT), malignant tumor, or primary immunodeficiencies to be independently associated with MRSA or *P. aeruginosa* isolation from respiratory cultures. Instead, our findings reinforce prior evidence that influenza, substance use disorder, and lung abscess/empyema are associated with MRSA, whereas bronchiectasis and COPD are associated with *P. aeruginosa*.^11–16^ These findings are reassuring for ASPs at centers with large transplant and oncology populations where optimizing use of BSAs is often challenging.

Our study adds to a limited but growing body of evidence that immunocompromising conditions are not independently associated with MRSA or *P. aeruginosa* in CAP.^17,18^ A secondary analysis of the Global Initiative for MRSA Pneumonia (GLIMP) database found that, while *P. aeruginosa* CAP was more prevalent among immunocompromised patients, immunocompromise was not a risk factor after multivariate adjustment for confounders.^17^ In a separate observational study of moderately immunocompromised patients who did not meet IDSA/ATS criteria for BSAs, no mortality difference was found between those treated with standard versus BSA.^18^ Both of these studies evaluated immunosuppressive therapies, such as corticosteroids, in addition to immunocompromised conditions, such as malignant tumor and transplantation.

We observed a higher prevalence of *P. aeruginosa* (5.5%) compared with prior studies (1.2–2.1%), likely reflecting selection for a sicker cohort due to the requirement for respiratory cultures.^18–20^ This is supported by a high ICU admission rate (41.6%). In studies with high rates of respiratory cultures (>85%), *P. aeruginosa* has been reported to be around 4%.^17,21^ Notably, the isolation of MRSA or *P. aeruginosa* in respiratory culture was not associated with ICU admission in a secondary analysis. Collectively, these findings suggest that immunocompromising conditions alone may be insufficient to guide empiric BSAs in CAP.

Two unexpected findings were that PUD was associated with both MRSA and *P. aeruginosa*, and liver disease was inversely associated with *P. aeruginosa*. Observational data have demonstrated associations between acid-suppressive therapies and pneumonia, as well as increased intestinal colonization with multidrug-resistant organisms (MDROs).^22,23^ However, an association of acid-suppression with MDROs, including *P. aeruginosa* and MRSA, within the context of CAP has not been evaluated. Given that we did not capture whether patients were on acid-suppressive therapies, these findings would need to be formally evaluated as the association likely reflects residual confounding. For liver disease, chronic fluoroquinolone exposure could theoretically influence *P. aeruginosa* CAP risk. However, we did not capture whether these patients were on spontaneous bacterial prophylaxis.

Our study has some important limitations. First, we relied on ICD-10 codes and the CCI to identify immunocompromising conditions. This limited our ability to evaluate all the conditions qualifying for immunocompromise in CAP as outlined by CHEST, specifically those related to pharmacotherapies.^24^ These pharmacotherapy-related conditions were evaluated in the studies by Di Pasquale and Saravolatz and were not found to be independently associated with MRSA or *P. aeruginosa,* as previously mentioned.^17,18^ However, with increased use of immunosuppressing agents since those reports, this merits further study. Since we did not capture antimicrobial exposures, we are unable to comment on the utilization or appropriateness of BSAs within our cohort. We also did not capture the timing of CAP hospitalization relative to date of transplantation as this may influence infection risk profiles. Our findings may not be generalizable to other institutions, as our cohort had a high ICU admission rate—likely reflecting higher acquisition of respiratory cultures, which are more commonly obtained in the ICU setting. Additionally, we did not stratify our analysis to evaluate temporal fluctuations in respiratory viruses, such as influenza or COVID-19. However, neither respiratory virus was found to have a significant association with either MRSA or *P. aeruginosa* pneumonia besides influenza with MRSA pneumonia [aOR 7.64]. Lastly, since we included only patients who had respiratory cultures, we may have missed patients with MRSA or Pseudomonas CAP who did not have respiratory cultures obtained.

In conclusion, our results support prior studies that did not find immunocompromising conditions to be associated with MRSA or *P. aeruginosa* in patients hospitalized with CAP. Our results affirm previously demonstrated risk factors for MRSA and *P. aeruginosa* in CAP and add additional confidence that immunocompromising conditions evaluated in this cohort were not associated with MRSA or *P. aeruginosa* CAP. Overall, these findings support an individualized approach to empiric CAP therapy, prioritizing established clinical risk factors over immunocompromised status alone.

## Supporting information

10.1017/ash.2026.10849.sm001 Mallory et al. supplementary material Mallory et al. supplementary material

## Supplementary material

The supplementary material for this article can be found at https://doi.org/10.1017/ash.2026.10849

## Table 1. Long description

A table summarizing patient population characteristics. The table has 40 rows and 2 columns. The first column lists various characteristics, and the second column provides corresponding values. Row 1: Age at admission median (IQR), 68 (57, 77). Row 2: Male sex, n (%), 916 (61.7%). Row 3: BMI median (IQR), 25.7 (22.3, 30). Row 4: Race, n (%): American Indian/Alaskan Native, 18 (1.2%). Row 5: Asian, 49 (3.3%). Row 6: Black or African American, 50 (3.4%). Row 7: Hispanic or Latino, 128 (8.6%). Row 8: Other, 11 (0.7%). Row 9: White, 1,229 (82.8%). Row 10: Congestive heart failure, 475 (32%). Row 11: Peripheral vascular disease, 653 (44%). Row 12: Cardiovascular disease, 265 (17.8%). Row 13: Diabetes mellitus, 358 (24.1%). Row 14: Renal disease, 462 (31.1%). Row 15: Liver disease, 328 (22.1%). Row 16: COPD, 554 (37.3%). Row 17: Bronchiectasis, 108 (7.3%). Row 18: Lung abscess / empyema, 32 (2.2%). Row 19: PUD, 77 (5.2%). Row 20: Rheumatologic disease, 109 (7.3%). Row 21: Hemiplegia, 49 (3.3%). Row 22: Dementia, 51 (3.4%). Row 23: Cancer, 568 (38.2%). Row 24: Metastatic solid tumor, 244 (16.4%). Row 25: AIDS, 11 (0.7%). Row 26: Primary immune deficiency diseases, 425 (28.6%). Row 27: Hypogammaglobulinemia, 57 (3.8%). Row 28: Alcohol use disorder, 82 (5.5%). Row 29: Substance use disorder, 61 (4.1%). Row 30: Tobacco use, 32 (2.2%). Row 31: COVID-19, 129 (8.7%). Row 32: Influenza, 35 (2.4%). Row 33: Any transplant, 240 (16.2%). Row 34: HSCT, 70 (4.7%). Row 35: Any SOT, 173 (11.6%). Row 36: Heart transplant, 47 (3.2%). Row 37: Liver transplant, 41 (2.8%). Row 38: Kidney transplant, 107 (7.2%). Row 39: Pancreas transplant, 5 (0.3%). Row 40: Multiple SOT, 27 (1.8%).

Navigate back to Table 1..

## Table 2. Long description

A table comparing risk factors for patients with MRSA pneumonia. The table has 5 rows and 6 columns. The columns are labeled: Characteristic, Unadjusted OR (95% CI), Unadjusted P-value, Adjusted for age, sex, BMI OR (95% CI), Adjusted for age, sex, BMI P-value, Adjusted for age, sex, BMI and significant risk factors OR (95% CI), Adjusted for age, sex, BMI and significant risk factors P-value. Row 1: Lung abscess / empyema, 5.05 (1.56-16.32), .007, 4.22 (1.20-14.86), .025, 4.58 (1.15-18.19), .031. Row 2: PUD, 3.08 (1.10-8.66), .033, 2.84 (0.96-8.44), .060, 4.09 (1.29-12.94), .017. Row 3: Alcohol use disorder, 3.13 (1.21-8.11), .019, 2.85 (0.87-9.27), .082. Row 4: Substance use disorder, 5.49 (2.21-13.63), <.001, 5.45 (1.91-15.60), .002, 8.29 (2.55-26.97), <.001. Row 5: Influenza, 6.31 (2.18-18.25), <.001, 5.58 (1.82-17.08), .003, 7.64 (2.01-29.09), .003.

Navigate back to Table 2..

## Table 3. Long description

A table comparing risk factors for patients with P. aeruginosa pneumonia. The table has 6 rows and 7 columns. The columns are labeled: Characteristic, Unadjusted OR (95% CI), Unadjusted P-value, Adjusted for age, sex, BMI OR (95% CI), Adjusted for age, sex, BMI P-value, Adjusted for age, sex, BMI and significant risk factors OR (95% CI), Adjusted for age, sex, BMI and significant risk factors P-value. The rows are labeled: Renal disease, Liver disease, COPD, Bronchiectasis, Lung abscess / empyema, PUD. Row 1: Renal disease, 0.55 (0.32-0.97), 0.040, 0.53 (0.30-0.95), 0.033, 0.63 (0.33-1.19), 0.153. Row 2: Liver disease, 0.40 (0.19-0.83), 0.014, 0.41 (0.19-0.88), 0.022, 0.39 (0.17-0.92), 0.030. Row 3: COPD, 3.02 (1.82-5.01), <.001, 2.84 (1.69-4.77), <.001, 2.60 (1.34-5.05), 0.005. Row 4: Bronchiectasis, 6.43 (3.76-11.01), <.001, 5.11 (2.86-9.13), <.001, 3.67 (1.85-7.28), <.001. Row 5: Lung abscess / empyema, 2.58 (0.93-7.19), 0.070, 2.55 (0.85-7.62), 0.094, 1.72 (0.47-6.36), 0.415. Row 6: PUD, 2.70 (1.31-5.57), 0.007, 2.50 (1.18-5.30), 0.017, 3.48 (1.44-8.44), 0.006.

Navigate back to Table 3..
