# Supplementary material for: Risk factors for methicillin-resistant Staphylococcus aureus and Pseudomonas aeruginosa community-acquired pneumonia at a high-volume transplant center
Source: Antimicrob Steward Healthc Epidemiol. 2026 Sep 9;6(1):e274. doi: 10.1017/ash.2026.10849 (PMC13760419; doi:10.1017/ash.2026.10849)
Supplement: Supplementary file 1 [file S2732494X26108493sup001.docx]

**Table 1**. Complete analysis of risk factors for methicillin-resistant *Staphylococcus aureus* community-acquired pneumonia

| Characteristic | Unadjusted | | Adjusted for Age, Sex, BMI | | Adjusted for Age, Sex, BMI and Significant Risk Factors | |
| --- | --- | --- | --- | --- | --- | --- |
|  | OR (95% CI) | p-value | OR (95% CI) | p-value | OR (95% CI) | p-value |
| Congestive heart failure | 0.88 (0.40 - 1.92) | 0.744 | 0.87 (0.38 - 1.98) | 0.737 | -- | -- |
| Peripheral vascular disease | 0.79 (0.37 - 1.65) | 0.525 | 0.78 (0.36 - 1.69) | 0.534 | -- | -- |
| Cardiovascular disease | 0.94 (0.37 - 2.40) | 0.898 | 0.89 (0.33 - 2.40) | 0.815 | -- | -- |
| Diabetes | 0.95 (0.41 - 2.20) | 0.901 | 0.92 (0.38 - 2.22) | 0.859 | -- | -- |
| Renal disease | 0.78 (0.35 - 1.74) | 0.542 | 0.76 (0.33 - 1.75) | 0.523 | -- | -- |
| Liver disease | 0.88 (0.37 - 2.13) | 0.780 | 0.82 (0.33 - 2.06) | 0.675 | -- | -- |
| COPD | 0.79 (0.37 - 1.71) | 0.556 | 0.77 (0.35 - 1.70) | 0.522 | -- | -- |
| Bronchiectasis | 1.78 (0.64 - 4.94) | 0.270 | 1.57 (0.51 - 4.83) | 0.432 | -- | -- |
| **Lung abscess / empyema** | 5.05 (1.56 - 16.32) | 0.007 | 4.22 (1.20 - 14.86) | 0.025 | **4.58 (1.15 - 18.19)** | **0.031** |
| **PUD** | 3.08 (1.10 - 8.66) | 0.033 | 2.84 (0.96 - 8.44) | 0.060 | **4.09 (1.29 - 12.94)** | **0.017** |
| Rheumatologic disease | 1.05 (0.28 - 3.91) | 0.939 | 0.84 (0.19 - 3.60) | 0.811 | -- | -- |
| Hemiplegia | 1.42 (0.27 - 7.51) | 0.683 | 1.00 (0.13 - 7.51) | 0.997 | -- | -- |
| Dementia | 2.40 (0.64 - 9.04) | 0.197 | 2.05 (0.45 - 9.31) | 0.354 | -- | -- |
| Cancer | 0.55 (0.25 - 1.24) | 0.149 | 0.53 (0.23 - 1.23) | 0.139 | -- | -- |
| Metastatic solid tumor | 0.41 (0.11 - 1.53) | 0.185 | 0.34 (0.08 - 1.43) | 0.140 | -- | -- |
| AIDS | 1.98 (0.11 - 34.36) | 0.640 | N/A* | N/A* | -- | -- |
| Primary immunodeficiency disorders | 0.59 (0.26 - 1.36) | 0.213 | 0.51 (0.21 - 1.22) | 0.129 | -- | -- |
| Hypogammaglobulinemia | 2.67 (0.85 - 8.42) | 0.093 | 2.31 (0.68 - 7.88) | 0.182 | -- | -- |
| Alcohol use disorder | 3.13 (1.21 - 8.11) | 0.019 | 2.85 (1.02 - 7.95) | 0.046 | 2.85 (0.87 - 9.27) | 0.082 |
| **Substance use disorder** | 5.49 (2.21 - 13.63) | <0.001 | 5.45 (1.91 - 15.60) | 0.002 | **8.29 (2.55 - 26.97)** | **<0.001** |
| Tobacco use | 0.59 (0.04 - 9.81) | 0.710 | N/A* | N/A* | -- | -- |
| COVID-19 | 1.67 (0.61 - 4.59) | 0.319 | 1.56 (0.53 - 4.54) | 0.418 | -- | -- |
| **Influenza** | 6.31 (2.18 - 18.25) | <0.001 | 5.58 (1.82 - 17.08) | 0.003 | **7.64 (2.01 - 29.09)** | **0.003** |
| Any transplant | 0.42 (0.11 - 1.53) | 0.187 | 0.31 (0.07 - 1.33) | 0.114 | -- | -- |
| HSCT | 0.30 (0.02 - 4.98) | 0.402 | N/A* | N/A* | -- | -- |
| Any SOT | 0.61 (0.17 - 2.25) | 0.460 | 0.47 (0.11 - 1.99) | 0.304 | -- | -- |
| Heart transplant | 0.46 (0.03 - 7.55) | 0.583 | N/A* | N/A* | -- | -- |
| Liver transplant | 2.94 (0.78 - 11.07) | 0.112 | 2.39 (0.55 - 10.42) | 0.246 | -- | -- |
| Kidney transplant | 0.60 (0.12 - 3.14) | 0.547 | 0.36 (0.05 - 2.71) | 0.322 | -- | -- |
| Pancreas transplant | 4.05 (0.22 - 74.82) | 0.347 | N/A* | N/A* | -- | -- |
| Multiple SOT | 2.57 (0.48 - 13.80) | 0.272 | 1.60 (0.21 - 12.39) | 0.654 | -- | -- |
| Neutropenia (ANC <500) | 1.58 (0.09 - 27.11) | 0.754 | N/A* | N/A* | -- | -- |
| Abbreviations: CI = Confidence Interval, OR = Odds Ratio  *Not estimable. | | | | | | |

**Table 2**. Complete analysis of risk factors for methicillin-resistant *Pseudomonas aeruginosa* community-acquired pneumonia

| Characteristic | Unadjusted | | Adjusted for Age, Sex, BMI | | Adjusted for Age, Sex, BMI and Significant Risk Factors | |
| --- | --- | --- | --- | --- | --- | --- |
|  | OR (95% CI) *^1^* | p-value | OR (95% CI) *^1^* | p-value | OR (95% CI) *^1^* | p-value |
| Congestive heart failure | 0.95 (0.57 - 1.57) | 0.839 | 0.87 (0.52 - 1.47) | 0.609 | -- | -- |
| Peripheral vascular disease | 1.41 (0.87 - 2.28) | 0.163 | 1.34 (0.82 - 2.21) | 0.245 | -- | -- |
| Cardiovascular disease | 1.45 (0.84 - 2.51) | 0.185 | 1.27 (0.72 - 2.24) | 0.413 | -- | -- |
| Diabetes | 0.69 (0.38 - 1.24) | 0.210 | 0.79 (0.43 - 1.44) | 0.437 | -- | -- |
| Renal disease | 0.55 (0.32 - 0.97) | 0.040 | 0.53 (0.30 - 0.95) | 0.033 | 0.63 (0.33 - 1.19) | 0.153 |
| **Liver disease** | 0.40 (0.19 - 0.83) | 0.014 | 0.41 (0.19 - 0.88) | 0.022 | **0.39 (0.17 - 0.92)** | **0.030** |
| **COPD** | 3.02 (1.82 - 5.01) | <0.001 | 2.84 (1.69 - 4.77) | <0.001 | **2.60 (1.34 - 5.05)** | **0.005** |
| **Bronchiectasis** | 6.43 (3.76 - 11.01) | <0.001 | 5.11 (2.86 - 9.13) | <0.001 | **3.67 (1.85 - 7.28)** | **<0.001** |
| Lung abscess / empyema | 2.58 (0.93 - 7.19) | 0.070 | 2.55 (0.85 - 7.62) | 0.094 | 1.72 (0.47 - 6.36) | 0.415 |
| **PUD** | 2.70 (1.31 - 5.57) | 0.007 | 2.50 (1.18 - 5.30) | 0.017 | **3.48 (1.44 - 8.44)** | **0.006** |
| Rheumatologic disease | 1.56 (0.74 - 3.28) | 0.243 | 1.32 (0.61 - 2.88) | 0.480 | -- | -- |
| Hemiplegia | 0.94 (0.26 - 3.41) | 0.920 | 0.80 (0.19 - 3.38) | 0.759 | -- | -- |
| Dementia | 1.73 (0.64 - 4.70) | 0.279 | 1.13 (0.38 - 3.33) | 0.824 | -- | -- |
| Cancer | 1.29 (0.80 - 2.08) | 0.294 | 1.20 (0.73 - 1.95) | 0.474 | -- | -- |
| Metastatic solid tumor | 1.05 (0.57 - 1.93) | 0.880 | 0.98 (0.52 - 1.82) | 0.941 | -- | -- |
| AIDS | 2.58 (0.46 - 14.51) | 0.283 | 2.66 (0.32 - 21.99) | 0.365 | -- | -- |
| Primary immunodeficiency disorders | 1.00 (0.61 - 1.65) | 0.996 | 1.08 (0.64 - 1.81) | 0.780 | -- | -- |
| Hypogammaglobulinemia | 1.01 (0.33 - 3.06) | 0.986 | 0.88 (0.27 - 2.90) | 0.831 | -- | -- |
| Alcohol use disorder | 0.27 (0.05 - 1.40) | 0.120 | 0.21 (0.03 - 1.53) | 0.123 | -- | -- |
| Substance use disorder | 0.65 (0.18 - 2.35) | 0.509 | 0.64 (0.15 - 2.81) | 0.558 | -- | -- |
| Tobacco use | 1.30 (0.35 - 4.83) | 0.696 | 1.07 (0.25 - 4.63) | 0.930 | -- | -- |
| COVID-19 | 1.05 (0.49 - 2.28) | 0.894 | 1.15 (0.51 - 2.57) | 0.735 | -- | -- |
| Influenza | 1.73 (0.56 - 5.35) | 0.342 | 1.44 (0.42 - 4.90) | 0.560 | -- | -- |
| Any transplant | 0.75 (0.39 - 1.45) | 0.391 | 0.87 (0.43 - 1.74) | 0.689 | -- | -- |
| HSCT | 1.17 (0.44 - 3.12) | 0.756 | 1.32 (0.46 - 3.78) | 0.608 | -- | -- |
| Any SOT | 0.75 (0.35 - 1.61) | 0.460 | 0.83 (0.37 - 1.86) | 0.650 | -- | -- |
| Heart transplant | 1.82 (0.67 - 4.93) | 0.240 | 2.30 (0.78 - 6.74) | 0.129 | -- | -- |
| Liver transplant | 0.20 (0.01 - 3.31) | 0.262 | N/A* | N/A* | -- | -- |
| Kidney transplant | 0.55 (0.19 - 1.65) | 0.289 | 0.53 (0.16 - 1.73) | 0.292 | -- | -- |
| Pancreas transplant | 1.56 (0.09 - 28.48) | 0.764 | N/A* | N/A* | -- | -- |
| Multiple SOT | 0.31 (0.02 - 5.08) | 0.410 | N/A* | N/A* | -- | -- |
| Neutropenia (ANC <500) | 0.59 (0.03 - 9.97) | 0.713 | N/A* | N/A* | -- | -- |
| Abbreviations: CI = Confidence Interval, OR = Odds Ratio  *Not estimable. | | | | | | |
